# Solitary diffuse-type giant neurofibroma arising from the sciatic nerve in a 13-year-old: A rare occurrence

**DOI:** 10.18632/oncoscience.640

**Published:** 2025-12-23

**Authors:** Reshmi Sultana, Suryadevara Sailaja

**Affiliations:** ^1^Department of General Surgery, Senior Resident, All India Institute of Medical Sciences, Bibinagar, Telangana 508126, India; ^2^Department of Surgical Oncology, Consultant Surgical Oncologist, Alluri Sitaram Raju Academy of Medical Sciences, Eluru, Andhra Pradesh, India

**Keywords:** pediatric neurofibroma, nerve sheath tumour, function sparing surgery, microsurgical tumor excision, Schwann cell tumor

## Abstract

Giant solitary neurofibromas involving deep peripheral nerves, particularly the sciatic nerve, are exceptionally rare in pediatric patients and pose unique diagnostic and surgical challenges. They often remain asymptomatic due to their deep intermuscular location, which delays detection until significant growth occurs. We report a 13-year-old male with a painless posterior thigh swelling, incidentally noticed while playing. MRI revealed a 12.8 × 6.6 × 3.8 cm lobulated intermuscular mass along the sciatic nerve. Tru-cut biopsy suggested a cellular neurofibroma. The patient underwent function-preserving excision under microscopic magnification, with careful dissection of the tumor from individual sciatic nerve fascicles. Intraoperative neurophysiological monitoring (IONM) was not available, highlighting the challenges of ensuring nerve preservation without real-time feedback. Postoperatively, transient foot drop occurred but resolved completely with physiotherapy. Histopathology confirmed a diffuse-type cellular neurofibroma with patchy S100 and SOX10 positivity, focal CD34 expression, and low Ki-67, supporting a benign profile. This case underscores the importance of meticulous surgical technique, the potential value of IONM, and vigilant postoperative care in achieving optimal outcomes for deep-seated pediatric neurofibromas.

## INTRODUCTION

Neurofibromas are benign tumors arising from the nerve sheath of the peripheral nervous system, capable of developing along the entire nerve pathway extending from the dorsal root ganglion to the terminal nerve branches. A neurofibroma that is not linked to neurofibromatosis type 1 is classified as a solitary neurofibroma [1]. Neurofibromas are classified into diffuse, plexiform, and localized types.

Solitary neurofibromas generally occur sporadically and more commonly involve superficial nerves. Giant solitary neurofibromas involving the deeper nerve like sciatic is rare, and its occurrence in paediatric age group makes it exceptionally uncommon. This rarity is largely attributed to the slow-growing nature of neurofibromas, which ideally require years to attain a significant size, making their presence uncommon in children. When such tumors do develop, they are often located in deep or anatomically permissive regions, allowing gradual expansion without early symptoms, which further delays detection. Published reports of solitary neurofibromas in deep or anatomically permissive sites in the pediatric age group are very limited, with some cases reported in the sacral region, thigh, neck, and scrotum [2–4].

## CASE REPORT

A 13-year-old male child presented to us with complaints of swelling in posterior aspect of right thigh for 4 months. The swelling was incidentally noticed while he was playing and has been gradually progressing since then. There weren’t any complaints of pain, paranesthesia, and features suggestive of impairment of limb function.

**Figure 1:**
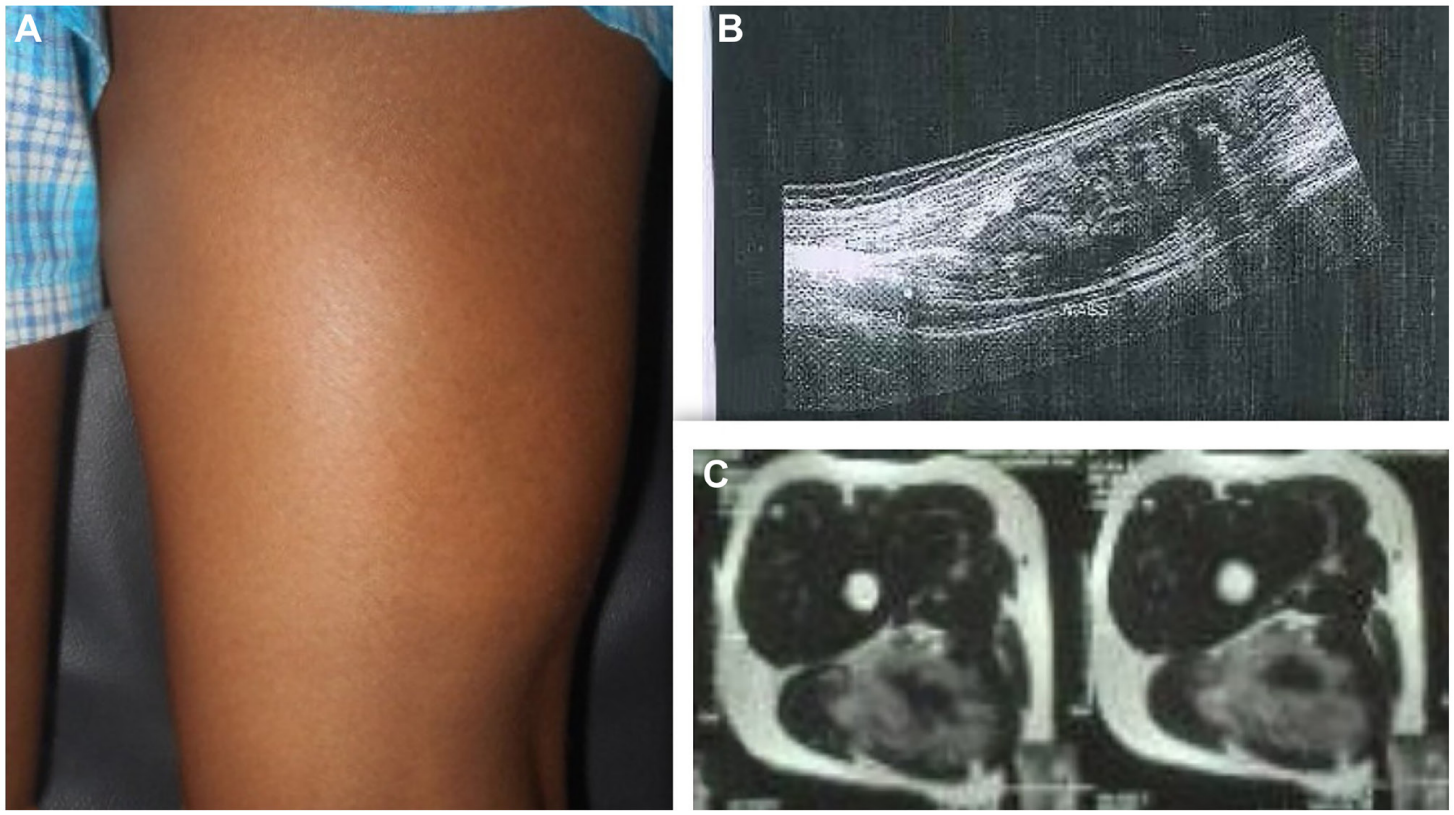
(**A**) Clinical picture, (**B**) Usg showing intramuscular heterogenous lesion arising from sciatic nerve, (**C**) MRI showing heterogeneously isointense mass measuring 12.8 × 6.6 × 3.8 cm with features suggestive of neurofibroma.

On examination, a smooth, firm and non-tender swelling of approximately 14 × 7 cm was palpated in the posterior aspect of right thigh. (Figure 1) Swelling was prominent on flexion of knee and was mobile in horizontal direction but with limited mobility in vertical direction. On systemic examination, there weren’t any features suggestive of neurofibromatosis- type 1.

**Figure 2:**
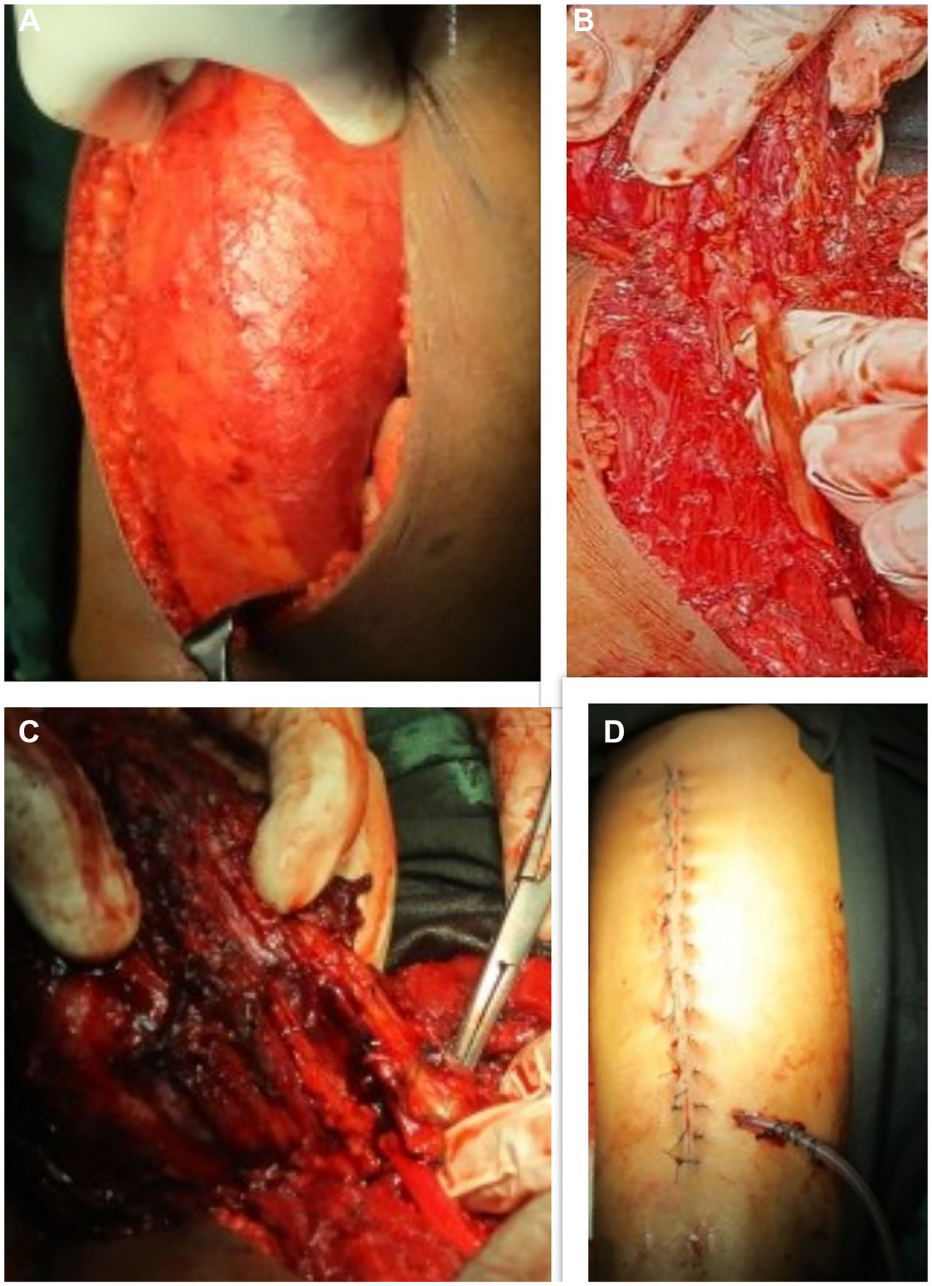
(**A**–**C**) Intraop images showing encasement of sciatic nerve, (**D**) Suture line with suction drain *in situ*.

Ultrasound showed an intramuscular heterogenous lesion arising from sciatic nerve. MRI revealed a large, lobulated, irregular T2 heterogeneously isointense mass measuring 12.8 × 6.6 × 3.8 cm within the intermuscular plane along the neurovascular bundle of the right mid-thigh, extending into the lower thigh. The mass involved the soft tissue compartment without evidence of intraosseous extension, findings suggestive of a neurofibroma. (Figure 1) Tru-cut biopsy showed a spindle-cell lesion with fascicular architecture, elongated tapering nuclei, and pale eosinophilic cytoplasm in a collagenous stroma with focal myxoid change. No atypia, necrosis, or significant mitoses were noted, and the features were consistent with a cellular neurofibroma. A function-preserving excision was performed with the patient in the prone position. The lesion was located in the intermuscular plane beneath the biceps femoris, densely adherent to surrounding musculature, and completely encasing the sciatic nerve. (Figure 2) Careful exposure of the sciatic nerve was achieved, and the nerve was meticulously traced along its course. Using surgical loupes for enhanced magnification, microscopic dissection allowed the tumor to be separated from individual fascicles without injury. The mass was excised completely while preserving the neural continuity. Histopathological analysis confirmed the lesion as a peripheral nerve sheath tumor, specifically a cellular neurofibroma of the diffuse type. Immunohistochemistry demonstrated patchy S-100 and positive SOX10 staining, confirming the neural origin of the tumor. CD34 showed focal positivity in the stroma, and Ki-67 was low, supporting a benign profile. EMA, desmin, and SMA were negative, excluding the possibility of perineurioma and smooth muscle tumour.

Patient developed foot drop in the immediate post operative period which gradually recovered with physiotherapy sessions without any residual disability.

## DISCUSSION

Peripheral nerve sheath tumors (PNSTs) are classified into schwannomas, neurofibromas, and malignant peripheral nerve sheath tumors. Schwannomas and neurofibromas are benign and together account for approximately 10% of all benign soft tissue tumors. Neurofibromas show no gender or racial predilection and can occur at any age, though isolated lesions are most common between 20 and 40 years. They typically measure less than 2 cm and are more frequently located in the head and trunk [5]. Based on anatomical location, neurofibromas are divided into superficial and deep types. Superficial neurofibromas lie between the skin and muscle fascia, presenting as palpable, soft nodules or papules with ill-defined margins, appearing as diffuse lesions on MRI, and rarely undergoing malignant transformation. Deep neurofibromas arise beneath the muscle fascia, often involve nerves, vessels, or visceral structures, are usually non-palpable, larger, symptomatic, and display a nodular or target-like pattern on MRI, with a higher risk of malignant transformation, particularly in patients with neurofibromatosis type 1 [6].

Although both schwannomas and neurofibromas are derived from Schwann cells, they differ in morphology and behavior. Schwannomas are generally well-encapsulated, circumscribed, characterized by elongated, whorled configurations of palisading nuclei. They may show cystic change, Antoni A and B areas, and Verocay bodies, with diffuse S100 positivity. Whereas neurofibromas lack encapsulation, demonstrate a more infiltrative growth pattern with variable cellularity and are composed predominantly of spindle cells embedded within a loose collagenous matrix, interspersed with fibroblasts, mast cells, and axons. Malignant peripheral nerve sheath tumors are aggressive, may arise from pre-existing neurofibromas, and show increased cellularity, mitoses, and necrosis, with focal S100 positivity and occasional INI-1 loss in epithelioid variants [7].

Computed tomography (CT) shows a well-defined mass with a density lower than that of muscle. On magnetic resonance imaging (MRI), neurofibromas are characteristically described as having low to isointense signals compared to muscle on T1-weighted images and appearing hyperintense on T2-weighted images [8].

Immunohistochemistry (IHC) is crucial in differentiating schwannomas, neurofibromas, and malignant peripheral nerve sheath tumors (MPNSTs). Schwannomas exhibit strong, diffuse S100 positivity and SOX10 expression, while neurofibromas show variable S100 expression due to their mixed cellular composition. In contrast, MPNSTs are characterized by reduced or focal S100 staining, loss of H3K27me3 trimethylation, and a high MIB-1 proliferation index, with occasional SOX10 positivity. These markers provide critical insights for accurate tumor classification and diagnosis [9].

While schwannomas and neurofibromas account for the majority of peripheral nerve sheath tumors, other soft tissue and nerve-related lesions must also be considered in the differential diagnosis. Perineuromas are benign tumors of perineural cells, characterized by EMA, Claudin1, and GLUT1 positivity and absence of S100 expression. Dermatofibromas consist of dermal fibroblasts and histiocytes with FXIIIA, CD163, and CD68 positivity, whereas. Dermatofibrosarcoma Protuberans is a locally aggressive fibroblastic sarcoma of the dermis and subcutis, exhibiting CD34 positivity and COL1A1-PDGFB fusion. Other important entities include superficial leiomyomas expressing SMA and Desmin, Neurotized Melanocytic nevi with melanocytic markers, ganglioneuromas demonstrating S100 in Schwann cells and synaptophysin in ganglion cells, Plexiform Fibrohistiocytic tumors with SMA positivity and S100 negativity, and Desmoplastic Melanomas, which show S100 and SOX10 positivity along with dermal scarring and cytologic atypia. Awareness of these histologic and immunohistochemical features is crucial for accurate diagnosis and distinction from PNSTs [10].

Function-sparing complete surgical excision remains the preferred treatment for neurofibromas, including deep-seated lesions encasing major nerves such as the sciatic nerve. This approach is technically demanding due to the lack of a distinct capsule and the risk of fascicular injury, making meticulous microsurgical dissection essential. The use of magnification and stimulating forceps allows careful separation of tumor tissue from functional fascicles. Intraoperative neurophysiological monitoring (IONM) further enhances safety, with triggered electromyography (EMG) and nerve action potentials (NAPs) providing real-time feedback on motor pathway integrity. While EMG facilitates mapping of innervated muscles, it can be influenced by anesthetic agents; NAPs, unaffected by muscle relaxants, are particularly useful in defining lesion margins and guiding precise dissection [11]. Historically, open excision without these adjuncts required extensive dissection and carried significant risks, including nerve injury, sensory deficits, motor weakness, neuropathic pain, and local recurrence. Advances such as microsurgical techniques, IONM, and minimally invasive or ultrasound-guided approaches have reduced morbidity and improved functional outcomes. Nevertheless, tumors encasing major nerves remain high-risk, with potential complications including transient or permanent motor/sensory deficits, neurapraxia, or neuropathic pain. In our case, adjuncts like IONM and intraoperative nerve stimulation could not be used due to unavailability and financial constraints, necessitating careful conventional microsurgical dissection to preserve nerve function.

Given the potential for recurrence and growth in deep-seated neurofibromas, regular clinical examination and imaging every 6–12 months for at least 2–3 years is recommended, particularly in pediatric patients [12].

## CONCLUSIONS

Diffuse-type neurofibroma of the sciatic nerve in children is exceptionally rare. Accurate preoperative diagnosis, careful microsurgical excision, and vigilant postoperative care enable complete recovery with minimal morbidity. This case highlights the importance of early recognition and meticulous surgical planning in achieving optimal functional outcomes.
